# A research-based inter-institutional collaboration to diversify the biomedical workforce: ReBUILDetroit

**DOI:** 10.1186/s12919-017-0093-6

**Published:** 2017-12-04

**Authors:** Jeanne M. Andreoli, Andrew Feig, Steven Chang, Sally Welch, Ambika Mathur, Gary Kuleck

**Affiliations:** 1 Department of Natural Sciences, Marygrove College, Detroit, MI 48221 USA; 2 Department of Biology, University of Detroit-Mercy, Detroit, MI 48221 USA; 3 Department of Chemistry, Wayne State University, Detroit, MI 48202 USA; 4 Department of Pediatrics, Wayne State University, Detroit, MI 48202 USA

## Abstract

**Background and purpose:**

Faced with decades of severe economic decline, the city of Detroit, Michigan (USA) is on the cusp or reinventing itself. A Consortium was formed of three higher education institutions that have an established mission to serve an urban population and a vested interest in the revitalization of the health, welfare, and economic opportunity in the Detroit metro region that is synergistic with national goals to diversify the biomedical workforce. The purpose of this article is to describe the rationale, approach, and model of the Research Enhancement for BUILDing Detroit (ReBUILDetroit) Consortium, as a cross-campus collaborative for students, faculty, and institutional development. The ReBUILDetroit program is designed to transform the culture of higher education in Detroit, Michigan by educating and training students from diverse and socio-economically disadvantaged backgrounds to become the next generation of biomedical researchers.

**Key program highlights:**

Marygrove College, University of Detroit Mercy, and Wayne State University established a Consortium to create and implement innovative, evidence-based and cutting-edge programming. Specific elements include: (1) a pre-college summer enrichment experience; (2) an inter-institutional curricular re-design of target foundational courses in biology, chemistry and social science using the Research Coordination Network (RCN) model; and (3) cross-institutional summer faculty-mentored research projects for ReBUILDetroit Scholars starting as rising sophomores. Student success support includes intentional and intrusive mentoring, financial support, close faculty engagement, ongoing workshops to overcome academic and non-academic barriers, and cohort building activities across the Consortium. Institutional supports, integral to program creation and sustainability, include creating faculty learning communities grounded in professional development opportunities in pedagogy, research and mentorship, and developing novel partnerships and accelerated pipeline programming across the Consortium. This article highlights the development, implementation and evolution of high-impact practices critical for student learning, research-based course development, and the creation of inter-institutional learning communities as a direct result of ReBUILDetroit.

**Implications:**

Our cross-institutional collaboration and leveraging of resources in a difficult economic environment, drawing students from high schools with a myriad of strengths and challenges, serves as a model for higher education institutions in large, urban centers who are seeking to diversify their workforces and provide additional opportunities for upward mobility among diverse populations.

## Background

**Fig. 1:**
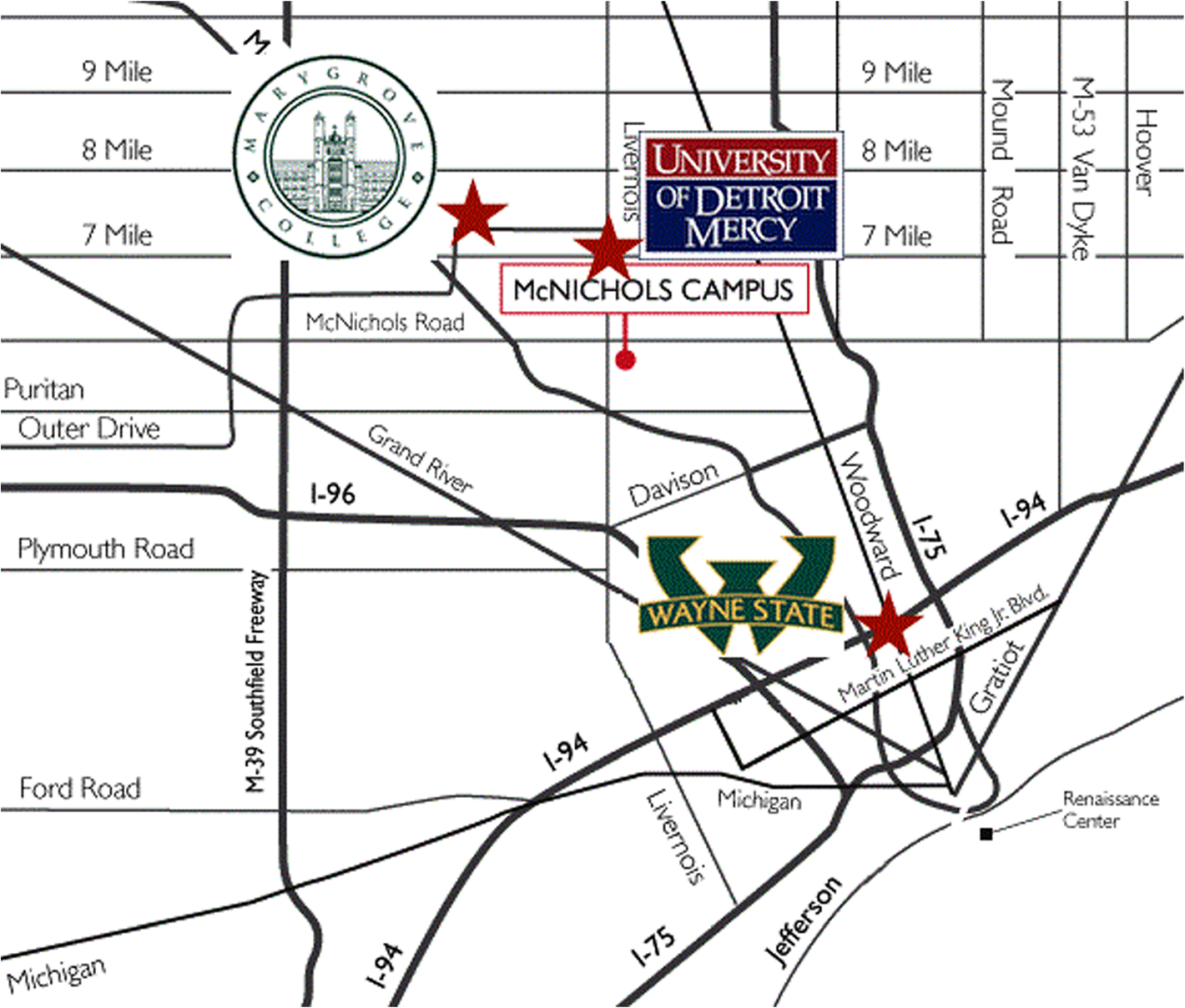
The ReBUILDetroit Consortium Institutions. Map Showing Relative Locations of Consortium Institutions in the ReBUILDetroit Program

After decades of economic decline, the city of Detroit is poised to re-emerge as the dynamic, resurgent economic powerhouse in Southeast Michigan, and consequently, the academic communities in and around the city must rise to the occasion by educating students from the area who have traditionally been neglected. Detroit-based institutions of higher education must lead in the *academic renaissance* of Detroit by assisting in the creation of a workforce that will support the large biomedical research community in Southeast Michigan by recruiting talented STEM students from underserved and socio-economically disadvantaged groups, using best practices to mentor and graduate them with advanced degrees. The three institutions that form the Research Enhancement for BUILDing Detroit (ReBUILDetroit) Consortium are *Marygrove College (private, PUI), University of Detroit Mercy (private, PUI), and Wayne State University (public, R-intensive [R1])* These institutions lie within a 5-mile radius to each other in Michigan’s most populous city, which is majority Black/African-American (82.7%)(Fig. 1) [1]. Collectively, the Consortium has an undergraduate population of approximately 22,000 students, of which 36% is minority enrollment and 85% of the undergraduate population is from the Metropolitan Detroit region.

The overarching goal of the ReBUILDetroit Program is to strengthen higher education practice to prepare students from underserved groups in metropolitan Detroit for a rigorous academic journey in pursuit of a biomedical degree and career, while striving to develop and nurture the self-confidence and personal acumen to be successful. Cognizant of the fact that most of these students will likely be facing personal challenges (e.g. level of academic preparedness, perception of self-efficacy and self-sufficiency), institutional issues (e.g. level of institutional cultural competency, transition to a learner-centered environment, elevating faculty mentorship etc.), and financial barriers [2–4], ReBUILDetroit is focused on addressing these challenges systematically and systemically for cohorts of students who enter as pre-freshmen through successful graduation with advanced biomedical degrees.

Historically, all three institutions have had a successful track record in securing funding in the area of undergraduate science education reform and creating pathways to biomedical research careers (Table 1). Further, each institution contributes a unique perspective from their cultural and academic ethos to the Consortium: two liberal arts PUI institutions whose missions are grounded in social justice and focus on excellence in pedagogical innovation and faculty-student interactions, and a research- intensive institution that is nationally recognized for its mentored undergraduate research experiences and biomedical graduate programs which train a large number of biomedical researchers who remain in the metropolitan Detroit region. The ReBUILDetroit program posits a new model of collaboration among higher education institutions to work closely together to expand, amplify and synergize these efforts to increase the enrollment, persistence and retention of underserved and socioeconomically disadvantaged students into biomedical research careers, which is the focus of the NIH BUILD Program.

**Table 1:** History of STEM Initiatives at ReBUILDetroit Consortium Institutions

School	Sponsor	Initiative	Highlights
Marygrove College	US Dept. of Education 2003–2008	Title III: Building the Capacity of Math and Science	Redesign of classrooms and laboratories
			Faculty-student research
			Professional Development
	US Dept. of Energy 2009	Strengthening the Capacity of Science & Mathematics Programs	Acquisition of research instrumentation for faculty-student research
	Project Kaleidoscope (PKAL) 2004–2007	“Science For All” Leadership Institution	Faculty Development
			Curricular Reform
	Kellogg Foundation 2014–2015	Building Our Leadership in Detroit (BOLD)	Leadership Training
			Participatory Action Research
			Experiential learning
University of Detroit Mercy	NSF 2014 - present	Advancement of Women in Academic Science and Engineering Careers (ADVANCE)	Professional Development
			Career support
			Institutional Transformation
	NSF 2016–2021	Scholarships for Robotics and Mechatronic Systems (S-STEM)	Scholarship support for students
	NSF: CCLI, TUES, ILI 1999–2003	UG Science/Engineering Education-(3 awards)	Curricular reform/development
Wayne State University	NIH 2011 - present	Initiative to Maximize Student Diversity (IMSD) Program	Student support
			Summer enrichment
			Mentored Research
	NSF 2013–2015	WSU-Widening Implementation and Dissemination of Evidence-based reforms (WSU-WIDER)	Faculty development to support adoption of evidence-based pedagogies in STEM
	NSF 2015–2020	Student Success Through Evidence-based Reforms (WSU-SSTEPs)	Faculty development to support adoption of evidence-based pedagogies in STEM
	NSF 2010 - present	Alliances for Graduate Education and the Professoriate (AGEP)	Mentoring
			Career Preparation
	NSF 2007–2010	Advancement of Women in Academic Science and Engineering Careers (ADVANCE)	Professional Development
			Career support
			Institutional Transformation
	Dept. of Education 2004 - present	McNair Scholars Program	Scholarship
			Mentored Research

Drawing on the collective strengths of each institution, the Consortium has created an innovative, dynamic and sustainable program that guides these students through a comprehensive, self-reinforcing educational experience that encourages development of the mindset of biomedical research experience necessary for the success of students in this career aspiration. The comprehensive design elements target students, faculty and institutional infrastructures. At the student level, the model incorporates pre-freshmen academic preparation for this rigorous journey, a Consortium-wide exposure to research methods and authentic, course-based undergraduate research experiences *as freshmen* and faculty-mentored summer research experiences throughout their undergraduate careers. Students are nurtured psycho-socially through elements that contribute to student success including intentional and intrusive mentoring, creating a cohort-based supportive learning community and career guidance throughout the program. Cross-institutional and institutional infrastructures support these student-centered program elements by (1) creating faculty learning communities grounded in professional development opportunities in pedagogy, research and mentorship; and (2) developing novel curricula, partnerships and pipeline programming across the Consortium, as well as engaging with the academic and industry biomedical workforce in the metropolitan Detroit area. It is anticipated that this tri-partite model (students, faculty, institutional) can serve as an exemplar for other urban institutions wishing to achieve similar aims.

**Fig. 2:**
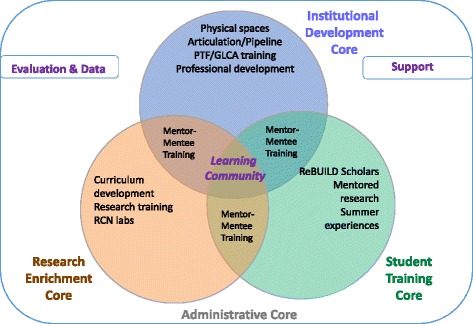
ReBUILDetroit Organizational Structure. Activities and aims of the Program are overseen by four Cores who collaborate across institutions and disciplines. This allows for synergies to develop among the Cores

Using a shared governance structure and coordinated activities from each of the four cores (Administrative, Institutional Development, Research Enrichment, and Student Training Cores) outlined in the NIH Building Infrastructure Leading to Diversity (BUILD) Initiative, we have initiated a cross -institutional teaching and learning community comprised of undergraduate students, pre-doctoral and post-doctoral students, faculty and staff. Our intentional design has created synergies among strategies and activities that lend to a collaborative, transformative and sustainable infrastructure to train the next generation of biomedical scientists in metropolitan Detroit (Fig. 2). This article highlights the theoretical framework and activities that shape the ReBUILDetroit Program’s achievement of its aims. Herein, we describe a broad implementation of a unique initiative and cross campus collaborative *in one city* and provide a strong rationale for our approach and model with evidence-based practices.

### The ReBUILDetroit model for preparing students for biomedical careers

The development of the ReBUILDetroit Program is based on best practices to recruit, retain and graduate students from underrepresented and socioeconomically disadvantaged groups into the biomedical research workforce. Published studies have demonstrated that addressing the social, academic and cultural biases embedded in higher education culture can have a profound impact on improving retention and graduation rates [5–12]. Barriers to success include academic and non-academic hurdles. These include, but are not limited to, poor academic preparation leading to a lack of self-efficacy, self-sufficiency and a sense of isolation; student financial constraints; institutional climates that are not conducive to assisting students and/or fostering inclusivity and cultural sensitivity; and limited knowledge of and limited access to research training opportunities [13–23]. Numerous studies have also demonstrated the efficacy of pre-college (before commencing the 1st year of school) summer bridge programs and authentic research experiences early in the student’s college education as foundational to improving student interest in science as a way of knowing, ownership of their own progress toward career research aspirations, and a profound sense of belonging to a scientific community [24–26]. Finally, results from other studies make a compelling argument for the value of mentoring as a formative, developmental experience for students and their progress toward graduation and is critical for increasing their capacity to overcome identified barriers [15, 27, 28].

The ReBUILDetroit Program coalesces these best practices into an integrative undergraduate experience in the context of an urban higher education setting using a number of cross-institutional strategies, including: (1) mentoring and intensive outreach beginning in the first year; (2) co-aligning and transforming the curriculum and pedagogy of select first year courses to make them inquiry-based, hands-on and engaging; (3) exposing students during the summer after their freshman year in authentic scientific/biomedical research with faculty mentors, and continue to engage them in research throughout their remaining undergraduate years; and (4) developing and sustaining a cross-institutional learning community for students beginning the summer before they enter their first year of the program through graduation. These strategies are expected to increase students’ confidence in their research skills, give them a sense of belonging in the scientific community, and make them feel like scientists/researchers. This, in turn, will increase persistence, graduation and entry into biomedical science research careers. So far, preliminary evidence suggests that these strategies are working.

- Three of the ten ReBUILDetroit Scholars attending the 2016 annual ABRCMS (Annual Biomedical Research Conference for Minority Students) won awards for best posters in the Social and Behavioral, Public Health categories. About the ABRCMS conference one student commented, “My view of research changed a lot after attending this conference. I see the bigger picture to research which I did not before. This experience was life changing!”
- Other ReBUILDetroit Scholars have presented their summer research at a disciplinary society conferences throughout the Midwest. About this experience, one student expressed increased feelings of belonging to the scientific community, commenting, “There were scientists of every race and ethnicity, gender and age. I saw researchers that looked like me….I am so thankful for being able to attend the conference. The experience brought me closer to my lab team and made me feel [like] a part of the larger scientific community.”
- Students also scored higher on a survey measuring perceptions of their research skills and science identity after completing their first-year curriculum, including the research methods course and Research Coordination (RCN) course. On the science identity scale, students raised their perceived rating from an average of 10.9 of 20 points before taking the course to an average of 16.8 after taking the course. Similarly, students increased their perceived rating from an average of 21.7 of 40 points before taking the course to an average of 35.3 on their research skills after the course, demonstrating the importance and relevance of our curriculum.

#### Outreach and early engagement

The ReBUILDetroit model includes recruiting students using a comprehensive evaluation of their qualifications. Preliminary outreach and early engagement of potential BUILD Scholars starts with the recruitment through Offices of Admissions at each institution who work closely with the Student Training Core (STC) to ensure eligibility requirements of students are met. ReBUILDetroit personnel partner with admissions staff to make additional contacts at local high schools, community colleges, and community events. Students can apply to the program online (rebuildetroit.org), or directly through the institution, where they indicate which program they are interested in and self-report demographic information including GPA, ACT or SAT scores, later verified by admissions staff. Students also write essays to demonstrate their interest in a research career and identify qualities of persistence and academic curiosity. Admissions personnel also flag general applicants to their institution who appear to meet the program requirements, reaching out with further information and encouraging them to apply to the program. A profile of each student is created that includes high-school transcripts, Federal Pell Grant eligibility (as a proxy for socio-economic status), GPA, ACT/SAT scores, race/ethnicity variables defined in the Notice of NIH’s Interest in Diversity [29], and the students’ essay responses. A ReBUILDetroit admissions team at each school reviews each student’s application and selects finalists to be interviewed. Applicants are tentatively accepted into the program, contingent on their attendance and performance in a 7-week Summer Enrichment Program (SEP) after which they are referred to as ReBUILDetroit Scholars. Table 2 summarizes the demographic profiles of the first two cohorts of ReBUILDetroit Scholars.

**Table 2:** Aggregated demographic profiles of the first two cohorts of ReBUILDetroit Scholars (2015–2017)

Institution	# of ReBUILDetroit scholars	Avg HS GPA	Avg ACT/SAT	% Pell Eligible^a^	% URGs^b^
UDM	43	3.5	25	81%	69%
MG	14	3.1	18	72%	77%
WSU	30	3.9	27	50%	97%

^a^Pell eligibility is the proxy used for determining low socioeconomic status

^b^URGs is self-reported and based on categories contained in the Notice of NIH’s Interest in Diversity [29]

The STC oversees an enhanced, pre-college summer enrichment experience to ensure students are adequately prepared for the rigors of academic transition in their freshmen year and exposed to other elements that will encourage student success (mentoring, faculty engagement, cohort building activities) [30]. This summer experience also initiates the development of an expansive, cross-institutional learning community critical to student intellectual development and important for the student’s sense of belonging.

#### Consortium-wide summer enrichment program (SEP)

The ReBUILDetroit SEP draws from each institution’s academic and cultural acumen in higher education, scientific research, and student development to deliver an integrative program that orients students into higher education and biomedical research within an urban context. Scholars are compensated to attend classes and workshops at their home institution that develop their scientific and academic skills, financial literacy, self-efficacy, and self-confidence in their aspirations to become a scientist. On select Fridays, each institution’s cohorts are brought together for Consortium-wide events, rotated among the institutions, thereby creating a larger learning community between institutions and increasing networking and cohort-building between students from the three institutions. The Friday Consortium activities are grounded in active and experiential learning which foster leadership and interpersonal communication skillsets; they have included residential weekends, service learning, urban field collection projects, and biomedical career development seminars. Further, as part of the multi-dimensional mentoring and building of learning communities, Scholars from the previous cohort interact with the new cohort of Scholars about their experiences during their first year of the program, promoting a sense of belonging and enhancing opportunities for peer-mentoring and networking [31, 32].

Student performance, including both academic and non-academic criteria, is evaluated throughout the SEP and at the conclusion of the SEP. Preliminary student evaluation results indicate a strong increase in normalized gain scores in students’ understanding of biomedical research (enhanced appreciation for the role of research in academia and society, sense of community with the biomedical research community and interest in post-graduate research) as well as their perceptions of experience with diversity (e.g., meeting people from diverse groups, opportunity to work cooperatively with different people). Evaluation results of the ReBUILDetroit SEP will be reported in a different article.

The friendships and sense of belonging to their home institution and the ReBUILDetroit community formed during the summer enrichment program is then intentionally fostered in the academic year with weekly institutional cohort meetings and bimonthly Consortium-level cohort meetings throughout the year. Additionally, the institutions have created a dedicated meeting/lounge space for the Scholars, further nurturing opportunities for development of the learning community and offering a safe space for peer support and peer mentoring. This comprehensive programming and support model across institutions to increase participants, peer networks, and opportunities for engagement in biomedical research would not be feasible if each institution worked independently.

#### Mentoring

Overcoming issues of attrition, especially during the first year, requires deliberate, consistent and persistent mentoring from a variety of student influences: faculty advisors, instructors and staff. One key aspect for retention and success of the ReBUILDetroit Scholars is the intrusive and intentional mentoring that they receive, supported by the weekly one-on-one meetings with an institution-specific Student Success Coordinator, who has extensive background in working with students and facilitating their adjustment to College. The Coordinator oversees the integration of student-focused advising and mentoring with consistent and persistent communication between the student, their academic advisor, instructors and the Student Success Coordinator***.*** It is the “high-touch” nature of the interaction between scholars and the Student Success Coordinator, first introduced by Earl [33], that allows for timely intervention to address academic and life issues [7, 34]. Interventions have included mandatory tutoring, required visits to faculty office hours, counseling, and more frequent meetings with the Student Success Coordinator. Further, this level of interaction has allowed for early intervention for dealing with the significant number of mental health issues on college campuses [35]. Additionally, peer mentoring activities strengthen the student’s capacity to overcome challenges and increases their self-confidence that they belong and can succeed. The costs associated with providing the human and physical infrastructure to deliver this multi-faceted approach to student support is justified in the first-year to second-year retention rates of the ReBUILDetroit Scholars (81%) compared to their peers.

### Undergraduate research engagement for ReBUILDetroit scholars

The three cores (Institutional Development Core, IDC; Research Enrichment Core, REC; Student Training Core, STC) also work collaboratively to facilitate the students’ introduction to research in their freshman year, in their transition to the summer research experiences in subsequent years and, ultimately, transitioning to graduate school and/or biomedical careers. This collaboration provides the keystone from which a multi-dimensional learning community is developed and built. While this learning community is in its beginning stages, it is grounded in peer and near-peer mentoring involving undergraduate students, pre-doctoral and post-doctoral students, faculty and staff and curricular and co-curricular innovations. Together, these programming elements outlined below, create a holistic undergraduate experience that develops and nurtures a biomedical researcher mindset for these undergraduates.

The Research Enrichment Core (REC) focuses on integrating research into sustainable curricular changes, particularly in the freshman year, across all three institutions. It does so using the Research Coordination Network model (RCN), whereby freshmen matriculate into a research methods course in the first semester and partake of a unique, discipline-specific authentic course-based research experience in their second term. These are mandatory courses that the Scholars must take in their first year, in addition to their regular classes. Every effort was made to align these courses with existing required foundational courses in the natural (biology, chemistry) and social (psychology, sociology) sciences so as to not create additional academic barriers to degree completion. These RCN courses, co-developed by disciplinary faculty across the Consortium, are academically intensive and introduce Scholars to the concept of what research is and what it entails, including occasional failure and uncertainty. ReBUILDetroit Scholars collect actual data and present their findings in poster presentations at the end of the academic year at a Consortium-wide event which is open to the public.

#### Research methods courses

The Research Methods Curriculum is designed to compare and contrast how different types of science contribute to biomedical research. Students are exposed to both quantitative and qualitative data and challenged to develop acumen in both areas. The course also works to facilitate communication between students and faculty through an interview project where students choose a faculty member from one of the Consortium schools and talk with them about their research and choice of discipline, and their motivation to select their research work. This project personalizes the interactions with faculty and breaks down important barriers that prevent some students from attending faculty office hours or developing the professional relationships with faculty that lead to better achievement in college [7, 36, 37]. During this semester, the ReBUILDetroit Scholars are tracked into one of three research streams; biology, chemistry or social science/health disparities in preparation for their RCN course the following semester.

A hallmark of this curricular design was the formation of an inter-institutional, interdisciplinary faculty learning community (FLC) who, through a backwards design process, worked together to delineate the desired student learning outcomes and core curriculum for the Research Methods course as well as the second-semester RCN courses (Table 3). Although common learning outcomes were mutually agreed upon, each institution had the flexibility to instill their mark in the courses based on the institutional context. For example, at UDM, faculty decided that separate research methods courses, dedicated to each discipline-based RCN course would be most beneficial. Marygrove College and Wayne State University created a universal Research Methods course that was open to ReBUILDetroit Scholars as well as other freshman STEM majors interested in advanced degrees in biomedical sciences.

**Table 3:** ReBUILDetroit Interdisciplinary Research Methods Common Course Student Learning Outcomes

1.Identify unsafe research practices	
2. Describe the ethical responsibilities of scientific researchers	
3. Identify and articulate a scientific question that has impact and relevance to society	
4. Apply the scientific method in various sub-disciplines to posed challenges	
5. Describe methods to collect, compare, contrast, analyze and interpret different types of data	
6. Communicate effectively in a variety of written and oral formats	
7. Use databases to search and access the scientific literature	
8. Work collaboratively with peers and develop a personal sense of accountability when working with teams	
9. Explain the role of the IRB in research oversight	

#### Research coordination network (RCN) courses

A centerpiece of the ReBUILDetroit curriculum is the Research Coordination Network (RCN) laboratories. These are course-based undergraduate research experiences (CUREs) *targeted at first year students*. CUREs were developed in three separate disciplines: Health Disparities, Biology and Chemistry representing the major directions from which a student might enter a biomedical research career. CUREs have been touted as one of the best ways to help scale the exposure to research [38–40]. Research projects in the ReBUILDetroit CUREs included work on the invertebrate life in the Clinton River watershed through the Barcode of Life project (Biology) [41], exploration of novel bacteriophage from Detroit soil samples through the HHMI-SEAPhages project (Biology) [42, 43], study of the biodistribution within garlic plants of heavy metal ions from contaminated soil (Chemistry), and mixed method studies of food choices or environmental health issues and its impact on urban health disparities (Psychology, Sociology, or Health Sciences depending on the campus). Regardless of the disciplinary content of the RCN courses, the overarching course goals were similar: (1) to provide an introduction to scientific skills and techniques related to a scientific discipline, and (2) to develop in students a sense of scientific identity within a scientific community.

The choice of the research themes for the RCN laboratories was intentional and deliberate as they address a range of real-world problems right in the Consortium’s “backyard” that can engage students, whereby learning becomes a social act. Such an engagement not only empowers the students to “own their learning”, thereby increasing their engagement and persistence with the subject, but it also increases students’ comprehension of abstract concepts and enhances their analytical and critical thinking skills necessary to address complex societal issues faced by the twenty-first century biomedical workforce. For example, in the Bar Code of Life RCN at Marygrove College, students collected live specimens from local waterways to determine species of the organism and then were able to upload their information into a national database, tying their work to local environmental and sustainability concerns.

The intent is that these projects are collaborative across the three campuses and thus model the RCN-Undergraduate Biology Education (UBE) program at NSF [44]. The goal is to develop and sustain a Consortium-wide CURE where each campus partner identifies a piece of the larger project that they wish to pursue and data from multiple classes can be stitched together to form a cohesive unit under the guidance of the FLC. This structure provides a couple of key elements of support. First and foremost, none of the faculty is alone in the research project, and thus tests a distributive collaboration model that allows dissemination of the project over multiple sites. Second, it provides a common educational experience for students from multiple campuses. Students have the ability to visit (in person or virtually) the other laboratory courses with whom they are collaborating and talk about the project as peers. This modality deepens student ownership of their project. Finally, it allows distributed use of infrastructure resources. Because it is a common project, it makes sense that the three institutions can share usage of the core facilities on the campus of the research partner. This allows faculty from the PUI institutions to develop connections with facility staff and gain expertise on the instrumentation in these facilities at the research partner institution.

Select quotes from the first cohort scholars were taken from qualitative interviews performed by external program evaluators. These quotes demonstrate the impact of RCN coursework on students’ academics and their understanding of biomedical research.

- “Our professors expected us to fail a few times before we actually found solutions. I failed many times and at first, I thought that was a bad thing. However, through this RCN, I have realized that failing is part of the process.”
- “RCN has helped me gain so much experience and knowledge as a researcher because we had a great instructor who was very knowledgeable and taught us many useful lessons to become a researcher.”
- “I was never sure how to explain these biomedical studies or how to work in this field in particular but I have gained a ton of confidence throughout this course.”

All of these projects are ultimately targeted at bringing students into faculty laboratories at the end of the first year of college. Having had the year-long curriculum with increasing exposure to research, the students are better prepared for the intensity of a research laboratory and what to expect from that experience, mitigating the initial culture shock of entering a laboratory. Our expectation is that the students will take greater ownership of their work and that they report greater satisfaction with the transition from knowledge consumer to knowledge generator as a result of the curricular innovations. Student outcomes from this first-year experience, relative to comparable non-BUILD scholars, are not yet available. To track the early Hallmarks of Success, we monitor Scholars’ science GPA, persistence within their majors, engagement in research on their respective campuses, and Scholars’ change in perceptions of science identity and research skills using a Retrospective Pre-Test design instrument.

#### Mentored summer research

Upon successful completion of their first year of college, including RCN coursework, Scholars are matched with faculty mentors across the three Consortium institutions to begin their undergraduate research careers. This process begins in second semester of their first year, where students and faculty submit their vitae electronically, and the matching is facilitated by structured, face-to-face meetings. This experience, at first, induced anxiety amongst the Scholars since they are simultaneously being interviewed and interviewing faculty. After the initial exposure to the process, the Scholars demonstrated increased self-confidence, were able to articulate their interest in the faculty member’s research and impressed the faculty with their development. All of the ReBUILDetroit Scholars were satisfactorily placed with many choosing a research disciplinary focus different from their initial intent.

Prior to the commencement of the 10-week long mentored research experience, research faculty and Scholars from all three schools are brought together to attend a week-long orientation that includes mentor training [36, 45], chemical safety and hygiene training, lab safety training and Responsible Conduct of Research (RCR) training. The end of this training week is marked by a lab coat ceremony that demarcates the Scholars’ transition into researchers. This is akin to the medical students’ white coat ceremony as they transition from pre-clinical to clinical training; a short, white lab coat is bestowed upon ReBUILDetroit Scholars, symbolic of their transition. The shared experience of orientation and ceremony are strategies that promote their entry into the larger scientific community, increases the scholars’ sense of self-identifying as a researcher, and promote persistence and retention of these scholars in the ReBUILDetroit program.

The mentored summer research experience culminates at a public Consortium-wide symposium where Scholars present their work at a poster presentation. Scholars are expected to continue their research in subsequent semesters and summers until graduation with either the same faculty mentor or different ones. Students are compensated for research and are expected to present their findings at local, state and national conferences, including ABRCMS (Annual Biomedical Research Conference for Minority Students) and SACNAS (Society for Advancement of Chicano and Native Americans in Science).

### The ReBUILDetroit model for developing faculty

Sustained organization and institution change in higher education requires multiple points of entry, or dimensions [46, 47]. Perhaps the most persistently challenging location for such change is faculty pedagogical practice [48–51]. The Institutional Development Core (IDC) of the ReBUILDetroit Program oversees the majority of activities related to faculty development which are grounded in a variety of theoretical frameworks that support sustainable and institutional transformation [52–57]. Integrative and dynamic faculty development opportunities addressing the learning infrastructure (curricular and co-curricular programming) and human infrastructure (collaborative learning communities) are the foundation of the ReBUILDetroit Faculty Development program.

Key features of the program include the formation of multi-dimensional learning communities – dynamic communities which span across institutions, disciplines and faculty ranks whereby ReBUILDetroit participants (ReBUILDetroit Scholars, Graduate Learning Community Advisors (GLCAs), Postdoctoral Teaching Fellows (PTFs), faculty members and research mentors become part of a Consortium-wide kaleidoscope network. These learning communities offer a structure for sustained dialogues, collegial support, peer mentoring, and act as an incubator for experimentation and innovation for participants [58–60].

One such dimension is the Research Coordination Network Faculty Learning Community (RCN FLC). Grounded in the experiential learning cycle [61], participants in the RCN FLC meet regularly over the course of the academic year. These meetings provide space to engage each other in understanding, adopting, implementing and assessing the best practices for the RCN course design model. They also allow regular reflection on and exploration of the social and cultural dynamics associated with success in diverse classrooms and laboratories. Through FLCs, participants better understand and support the contributions and challenges of colleagues across the three campuses, learn together the skills and best practices in multicultural undergraduate teaching and mentoring in the STEM disciplines, and enhance the exploration and production of high-quality science appropriate to their institutional context.

For example, since few of the faculty have worked in the space of Course-based undergraduate research, integral to the design and implementation of the Research Coordination Networks (RCNs), we have had to develop both processes and supports to help facilitate the adoption of these curricular reforms and activities. Campus cultures, with regard to teaching, service and scholarship, differ greatly across the Consortium and the resources that can be brought to bear are unequal. The placement of GLCAs and PTFs create parity across the institutions and serve several purposes. First, it creates another layer of mentoring to extend the personal, educational and professional growth of the Scholars [62–64]. Second, it extends the training of young professionals [54, 56, 62, 65, 66]. Third, when it works well, it provides classroom and content support for the primary instructor to make space and time for learning to teach in a more active and engaging manner through these project-based experiences. The teamwork necessary to make these projects succeed have brought faculty from these three institutions into close collaboration with each other, evidenced by recent inter-institutional presentations, grant submissions, and ReBUILDetroit sponsored pilot project proposals related to curriculum development and collaborative research projects. Such collaborations will strengthen research ties between institutions while enabling successful practices to be developed and shared across the Consortium.

The professional learning communities are supported through a robust array of faculty and staff professional development initiatives that are grounded by the explicit needs of the participants and lead to sustainable practices [51, 67–69]. Together, these are aimed to foster a praxis environment in which professionals from all three institutions can collaborate to enhance their pedagogical skill set, curriculum development, and knowledge, skills, and values related to multiculturally inclusive STEM education (rebuildetroit.org/faculty/professional-development/). To track the early Hallmarks of Success, we have designed a Retrospective Pre-Test design instrument to capture GLCA, PTF and faculty perceptions of changes in knowledge, attitudes and/or behaviors stemming from their participation in the learning communities and professional development programming.

### ReBUILDetroit: A novel model of inter-institutional transformation, sustainable institutionalization and propagation

In describing the barriers and opportunities for two-year and four-year STEM degree completion, the National Academies highlighted a collection of activities that support students to graduate with STEM degrees [6]. The ReBUILDetroit program is well aligned with the recommendations of this national report, of which early entry into authentic research experiences and the development of learning communities for scholars are prominent. Yet, promoting institutional transformation and cultural change is one of the most challenging and pervasive problems in higher education, including the ReBUILDetroit Consortium. It requires concerted effort and buy-in from both faculty and administration, and involves elements that pertain to the individual as well as to the collective environment of the institution. A shared vision is essential and all stakeholders must work synergistically to effectuate sustainable change. This shared vision is even more important when dealing with change in three institutions with different academic cultures, ethos and student demographics. These cultures are unified, however, in their commitment to serving the citizens of Detroit and increasing the diversity in the biomedical workforce.

The success of the ReBUILDetroit Program is predicated on building a strong, collaborative model of engagement between the three institutions, WSU as research partner and UDM and MG as primarily undergraduate institutions (PUIs). To strengthen collaboration, we have divided program responsibilities across the campuses to ensure engagement and create processes to integrate the work of faculty and staff to achieve our desired outcomes. The Student Training Core (STC), the Research Enrichment Core (REC) and the Institutional Development Core (IDC), work synergistically and collaboratively to guarantee joint supervision and integration of all Consortium activities; the Administrative Core provides the guidance and support for the entire program. Each core consists of a representative from each institution (faculty or staff) with one faculty champion from each institution chosen to be the Consortium lead for that Core. Thus, all three institutions have voice and authority in the deliberations in each Core. The Core leaders are empowered by the co-PIs to plan and create shared, joint policies for the Consortium. The co-PIs, in turn, consult with the Core leadership and reach a consensus on advocating for and implementing these policies on their individual campuses. Since all three co-PIs hold significant leadership positions, they have instituted changes which become part of the fabric of the academic landscape on their campus, leading to a shared vision of transformation with respect to the Program’s goals and which is essential for campus buy-in and program sustainability. This shared governance model of collaboration facilitates communication across the partner institutions, promotes mutual respect and accountability, and ensures that the needs of the individual partners as well as the Consortium are met.

**Fig. 3:**
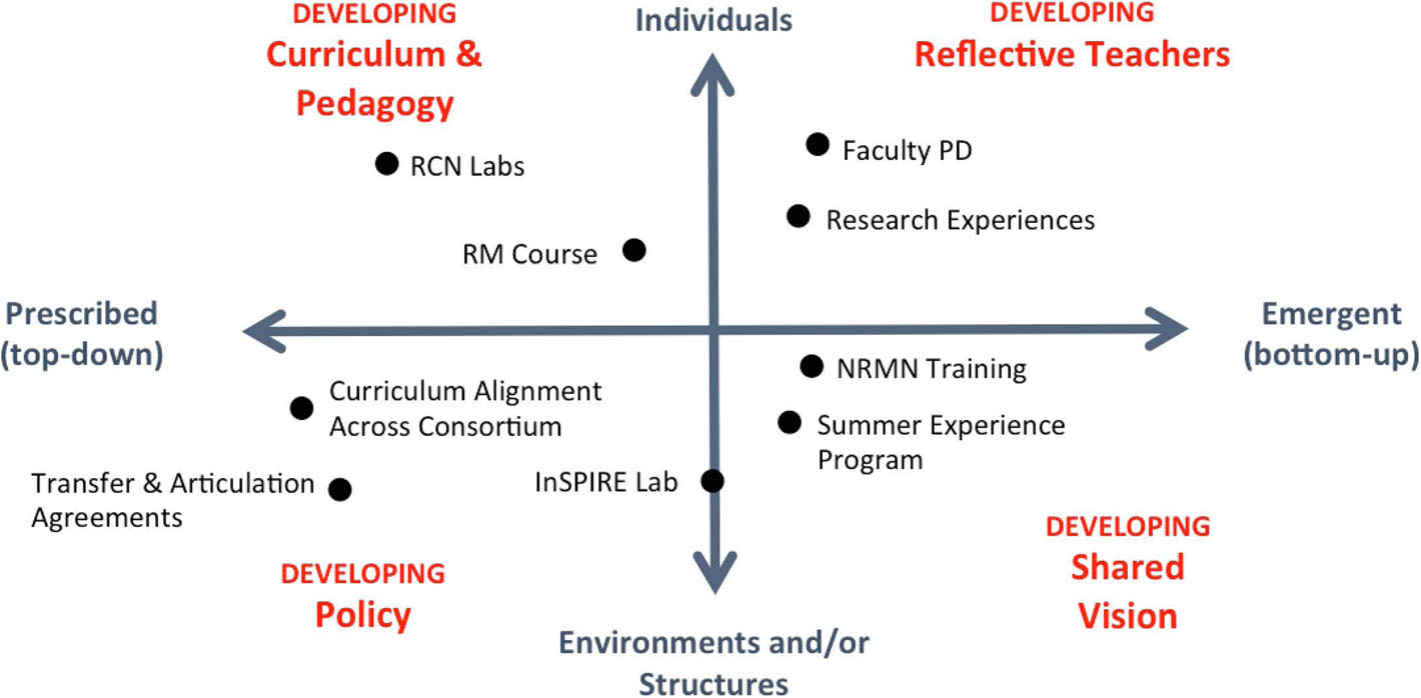
ReBUILDetroit Model of Institutional transformation. Mapping of ReBUILDetroit components to Henderson et al. [53] four-square model of institutional transformation

Another strength of the ReBUILDetroit program lies in its synergistic model for building and sustaining diversity in the biomedical research professions rather than focusing only on increasing access to supportive mechanisms to increase the pipeline of underrepresented groups into STEM fields. This tri-partite model of program activities (students, faculty, infrastructure) and combination of prescribed and emergent initiatives that encompass four different aspects of the ‘institution’ are key indicators of successful transformation and sustainable institutionalization [53] and lie at the core of the ReBUILDetroit program. These areas include developing curriculum and pedagogy, reflective teachers, policy, and shared vision (Fig 3).

Sustainability and institutionalization also necessitates that each Consortium partner, in their own way, adapts improvements developed through the ReBUILDetroit Program (e.g. mentoring, pedagogical innovation, research capacity) that become permanent aspects of the institution’s academic landscape. For example, each institution already had long established commitments to pedagogical innovation and undergraduate research; this grant has measurably strengthened those commitments and faculty engagement, *across all three institutions*. It has also sparked and invigorated faculty involvement with student research mentorship, increased interest in scholarly publications and created a dynamic faculty interest in collaboration in mentorship, pedagogy and research. The use of the CURE/RCN model, in particular, in the freshman year has spurred enthusiasm for reforming other laboratory-based classes at each institution to incorporate components that engage students in the scientific enterprise and improve student success. Another example is the new UDM iNSPIRE Laboratory which serves as a flexible, adaptable student research space and teaching laboratory serving all of the sciences, including the RCN labs [70, 71]. Moreover, it serves as a gathering space for faculty development through workshops and other training activities. Both ReBUILDetroit faculty and Scholars regard this space as a catalyst which energizes student and faculty involvement in research.

The geographic proximity (within 5 miles) of all three institutions affords unique partnerships, articulation agreements, research capacity and post-graduate training in biomedical fields. Its larger faculty size and greater research intensity not only allows WSU to serve as a hub for research experiences for a wider array of students but also as a conduit to spur inter-institutional faculty research collaborations. The proximity of the campuses allows faculty and students to commute between all three institutions within 15 min. This minimizes the burden of carving out time to engage in collaborative work. In addition, student mobility will be facilitated by plans for unique pipelines for students to pursue advanced degrees at UDM and WSU as part of the ReBUILDetroit Program. While each institution has detailed arrangements (transfer and articulation agreements) for students to transfer between institutions, the emergence of inter-institutional shared first year RCN curriculum and other activities are engendering the exchange of administrative best practices to streamline student mobility towards undergraduate degree completion and advanced degrees in biomedical sciences (e.g. undergraduate consortium agreements, 3 + 2 articulation agreements) [6, 23]. The enhancement of existing policies and procedures and the creation of new ones will institutionalize changes due to the ReBUILDetroit Program.

One aspect that we believe can be a model for future programs is the explicit inclusion of social science as a pathway to a biomedical career. In addition to faculty from the natural and health sciences, we have faculty from the social sciences engaged in the Summer Experience Program who have developed modules to introduce students to social science research in the context of biomedical research (i.e. using a health disparities model) as well as serving as faculty mentors in the Summer Research Experience. We believe this broadens the access for students to enter into biomedical research and may help retain students for whom the natural sciences is not the best fit, allowing for an alternative to the traditional STEM education pathway. The exposure to social sciences as a research discipline also aids the students in career discernment. Students are exposed to new career options that were previously unknown to them.

Many of the challenges we have faced are also our strengths. The institutional cultures of a large research institution and smaller, primarily undergraduate institutions create an opportunity for all of us to learn how higher education is approached at institutions with different mandates and cultures. In our collaboration, we have been compelled to work within the institutional cultures that exist, but also to work together to change our own institutional cultures; not to homogenize them, but to align and leverage them to better serve our students. We take the best of what we learn from each other and apply them to our own home institutions [72, 73].

Resources required for a program this size has been another challenge. While NIGMS/NIH funding has been crucial, succession planning and institutional commitment are also essential. As the program builds, increasing support staff that directly work with students is a necessary next step to ensure the success of our students and the program overall. Additional support needs to include co-curricular aspects of our program (food and housing, programming and activities) that are not explicitly covered by the funds awarded. Similarly, finding cost-effective and imaginative ways to expand faculty engagement, scaffold academic year research loading and collaborative pilot projects into faculty workloads and the tenure and promotion process, especially at the PUIs, is critical to achieving institutionalization [74]. Building and maintaining a multidimensional learning community is essential for the success of this program and we have been imaginative in finding ways to support these interactions, while also seeing how much more could be done with additional resources. To that end, each institution is challenged to leverage its unique support network of educational partners, organizations, community entities, and alumni to provide resources to expand and sustain the impact of the ReBUILDetroit Program.

It is anticipated that this ReBUILDetroit model can serve as an exemplar for other urban institutions wishing to achieve similar aims. While circumstances, unique to the institution and its milieu, will determine the actual steps required, critical elements must be in place for if this model was propagated at other institutions:

- Shared vision and mission by all institutions and stakeholders, founded on mutual respect, honesty and trust
- Organizational and administrative structures that support individual institutions and the Consortium as a whole
- Policy, process and communication plans to ensure accountability, shared governance and transparency
- Logic model that articulates assumptions, resources, activities, expected outcomes, and outputs
- Dynamic structures that allow for continuous improvement, adaptability and sustainable growth

We believe our contribution, nationally, is tied to the urban context of our program. All three schools are located in Detroit, a resilient city that has faced decades of economic decline, on the cusp of reinventing itself. All three schools have a mission to serve the people of Detroit and have a vested interest in its revitalization. Our successes in a difficult economic environment, drawing students from high schools with a myriad of strengths and challenges, and faced with a mature college-going employment market, serve as a model for schools in large, urban centers who are seeking to diversify their workforces and provide additional opportunities for upward mobility for its diverse populations.

## Abbreviations

ABRCMS: Annual Biomedical Research Conference for Minority Students
CURE: Course-based Undergraduate Research Experience
FLC: Faculty Learning Community
GLCA: Graduate Learning Community Advisor
GPA: Grade Point Average
HHMI: Howard Hughes Medical Institute
IDC: Institutional Development Core
MG: Marygrove College
NIH: National Institutes of Health
NRMN: National Research Mentoring Network
NSF: National Science Foundation
PTF: Postdoctoral Teaching Fellow
PUI: Primarily Undergraduate Institution
RCN: Research Coordination Network
RCR: Responsible Conduct of Research
ReBUILDetroit: Research Enhancement for BUILDing Detroit
REC: Research Enrichment Core
RPT: Retrospective Pre-Test
SACNAS: Society for Advancement of Chicano and Native Americans in Science
SEP: Summer Enrichment Program
STC: Student Training Core
STEM: Science Technology Engineering Mathematics
UBE: Undergraduate Biology Education
UDM: University of Detroit-Mercy
WSU: Wayne State University
